# Analytical Determination of Aflatoxin in Ground Corn Check Samples Completed by Multiple Laboratories over Several Years

**DOI:** 10.3390/foods13121918

**Published:** 2024-06-18

**Authors:** Ronald W. Sarver, Alexander T. Kostin, Benjamin F. Strong

**Affiliations:** Neogen Corp., Lansing, MI 48912, USA; akostin@neogen.com (A.T.K.); bstrong@neogen.com (B.F.S.)

**Keywords:** mycotoxins, analysis, contaminated food utilization, interlaboratory study

## Abstract

Mycotoxins are toxic molecules produced by multiple fungal species, including *Aspergillus* and *Fusarium*. Fungal infection of crops can result in mycotoxins entering the animal and human food supply. Enzyme-linked immunosorbent assays and other immunological assays have been developed to detect mycotoxins in foods. To calibrate the response of those methods, reference materials with known amounts of homogeneously dispersed mycotoxins are often utilized, where the mycotoxin concentrations have been determined using high-performance liquid chromatography coupled with absorbance or fluorescence detection methods, or high-performance liquid chromatography coupled with mass spectrometry detection methods. Therefore, it is important that the analytical methods provide accurate and precise quantitation of mycotoxins. The reference materials must also contain homogeneously dispersed known quantities of mycotoxin. To evaluate the accuracy and precision of mycotoxin reference materials and the analytical methods, quantitative results from multiple laboratories were completed each year for several years on ground corn check samples containing known levels of mycotoxins. Results for the quantitation of aflatoxin-containing corn reference samples are presented in this article.

## 1. Introduction

There are already many challenges to supplying safe and adequate amounts of food to meet human and animal needs. As population and demand for food increase, so does the need to increase the efficiency of food production and the need to remove unsafe food from the food supply. Sources of contaminated food can occur due to fungal infections of crops which under certain conditions can lead to formation of mycotoxins in the crops. These mycotoxins are toxic to humans and animals [1,2]. Fungal infection of crops adversely impacts areas of cropland leading to crop damage, decreased yields, contamination of the grain by mycotoxins, and animal and human disease. Several mycotoxins have been identified in corn, wheat, and other foods that adversely impact animal and human health including aflatoxins, fumonisins (B_1_, B_2_, and B_3_), zearalenone, ochratoxin A, deoxynivalenol, and trichothecenes [1,2,3,4,5]. Approximately 20 aflatoxins are known, with the subtypes B_1_, B_2_, G_1_, and G_2_ commonly found in food, and M_1_ and M_2_ which are found in human and animal milk. Chronic exposure to aflatoxins, which are toxic compounds, can lead to liver cancer in humans and death in cases of acute exposure [3,6]. Emerging mycotoxins continue to be closely examined for their effects on humans and animals [7,8].

Conditions which promote fungal infection of crops are expected to increase in geographical areas where fungal infections were not previously as common [9,10,11]. Computational models predict increases in aflatoxin B_1_ contamination in maize in Europe as a result of increasing average temperature [12]. Fortunately, enzyme-linked immunosorbent assays (ELISA) and other immunological-based assays which are amenable to screening grain for the presence of toxins have been developed to detect mycotoxins [13,14]. These assays often depend on the analysis of check samples or reference materials containing known amounts of mycotoxins as determined by high pressure liquid chromatography (HPLC) and ultra-performance liquid chromatography/mass spectrometry (UPLC/MS) methods [15,16,17]. Therefore, the accuracy and precision of the HPLC and UPLC/MS methods are critical to establishing the accuracy of the ELISA and other immunological-based assays. Reference materials must also contain accurately known and homogeneously distributed mycotoxin to be useful for evaluation of the methods. To assess accuracy, precision, reproducibility, and long-term trends in quantitative results for the determination of aflatoxin in ground corn using HPLC and UPLC/MS methods, a multi-laboratory study was conducted over multiple years using aflatoxin-containing ground corn check samples. 

## 2. Materials and Methods

### 2.1. Reference Materials

The following reference materials were purchased from Trilogy Analytical Laboratory (Washington, MO, USA); ground corn containing 4.8 ± 1.0 ppb total aflatoxin (Lot A-C-2252), 4.6 ± 0.5 ppb total aflatoxin (Lot 121129(4.6B)), 19.2 ± 2.0 ppb total aflatoxin (Lot A-C-2248), 21.8 ± 2.8 ppb total aflatoxin (Lot 121126(21.8B)) and 87.9 ± 11.9 ppb total aflatoxin (Lot A-C-2245, also designated Lot 121114(87.9)). The total aflatoxin concentration is determined by measurement of the sum of aflatoxin B_1_, B_2_, G_1_, and G_2_. In 2019 and 2020, a liquid standard spiked with 25 ng/mL aflatoxin B_1_ was also provided for analysis. In 2021, 2022, and 2023, a liquid standard spiked with 1000 ng/mL aflatoxin B_1_ was also provided for analysis.

Over all the years of the study, from 2019 through 2023, nine laboratories with established mycotoxin methods participated in this evaluation, although not all labs participated each year. Each laboratory that participated was sent one shipment of samples containing 9 double-blind coded samples with triplicate samples at each toxin level, as well as a vial of liquid aflatoxin stock. In the years 2019 through 2022, the 9 samples were composed of three 50-g aflatoxin reference material corn samples containing 4.8, 19.2, and 87.9 ppb total aflatoxin. In 2023, the 9 samples were composed of three 50-g aflatoxin reference material corn samples containing 4.6, 21.8, and 87.9 ppb total aflatoxin.

### 2.2. Laboratory Analytical Methods

Laboratory #1 used the Association of Official Analytical Collaboration (AOAC) International official method of analysis (OMA) 994.08 [18] for the determination of aflatoxins. Neogen’s Analytical Laboratory (Laboratory #2 in this report) analyzed samples using a Thermo Scientific Ultimate 3000 HPLC with Fluorescence Detection (FLD) on a FLD-3100 fluorescence detector with a dual-photomultiplier tube (Thermo Scientific, Waltham, MA, USA). Samples were prepared for analysis by extracting 25 g of sample with 125 mL of 70% methanol/water. The mixture was blended for 2 min, and 20 mL of filtered extract was diluted into 40 mL of phosphate buffered saline (PBS). If necessary, the extract was adjusted to pH 6 to 8, filtered through a glass fiber filter, and 15 mL of the diluted extract was added to an immunoaffinity column (Neocolumn for Aflatoxin DR, Neogen, Lansing, MI, USA). Bound aflatoxins were eluted from the immunoaffinity column with 1 mL of methanol and 1 mL of HPLC grade water. Samples (20 µL) were injected onto a Waters Sunfire C18 analytical column (5 μm particle size) held at 40.0 °C. The mobile phase was 60:20:20 water: acetonitrile: methanol with a flow rate of 1 mL/min. A Kobra cell (r-Biopharm, Darmstadt, Germany) was used to brominate the aflatoxin, and fluorescence was measured using 360 nm excitation and 440 nm emission.

The Federal Grain Inspection Services (FGIS) Technology and Science Division laboratory of the United States Department of Agriculture (Laboratory #3 in this report) analyzed samples using an Acuity UPLC/FLR, (Waters Corp., Milford, MA, USA). Samples were prepared for analysis by extracting 50 g of sample with 100 mL of 90% acetonitrile/water. The mixture was shaken for 30 min at 200 rpm. Then, 250 µL of filtered extract was mixed with 4.75 mL of 0.5% Tween 20 in water. The diluted extract was added to an immunoaffinity column (AflaTest WB, Vicam, Milford, MA, USA). Bound aflatoxins were eluted from the immunoaffinity column with 1.5 mL of methanol and diluted with 4.5 mL of water. Samples (20 μL) were injected onto a Waters Acquity BEH C18 analytical column (1.7 μm particle size) held at 40.0 °C. The mobile phase was 60:30:10 water: methanol: acetonitrile with a flow rate of 0.4 mL/min. Fluorescence was measured using 365 nm excitation and 445 nm emission.

Laboratories 4, 6, 7, and 9 extracted 25 g samples with 100 mL of 90% acetonitrile/water. Laboratories 4 and 6 diluted the extracts 1:5 in water and injected the diluted samples onto a HPLC-MS/MS to quantitate aflatoxins. Laboratories 7 and 9 added diluted extracts to immunoaffinity columns (AflaTest WB, Vicam, Milford, MA, USA). Extracts were eluted with methanol and then diluted 1:5 in water, followed by injection onto HPLC/FLD for the determination of aflatoxins. Laboratory 5 used AOAC OMA 2005.08 [18] where samples are extracted in 70% methanol, followed by dilution with water and immunoaffinity column cleanup. The samples were derivatized using a photochemical reactor for enhanced detection (PHRED) and analyzed on a HPLC-FLD. Laboratory #8 used modified International Organization for Standardization (ISO) methods to quantitate aflatoxins [19,20].

### 2.3. Statistical Analysis

Average results, standard deviation of the population (SD), and relative standard deviation (RSD) were calculated using equations provided in Microsoft 365 Excel. Z-scores were calculated using the average results for each lab minus the average result for all the laboratories divided by the SD of results for all the laboratories, z = (x − µ)/σ, where z = Z-score, x = average lab result, µ = average result from all the laboratories, and σ = SD of results from all the laboratories. Analysis of variance (ANOVA) of the results was completed using the one-way ANOVA hypothesis function available in Minitab 18 software (Minitab, LLC., State College, PA, USA.). Figures with experimental results, mean, and 95% confidence intervals were also generated using Minitab 18 software. 

## 3. Results

### 3.1. Analytical Results for 4.8 ppb and 4.6 ppb Aflatoxin-Containing Ground Corn Check Samples

Table 1 shows the results each laboratory obtained for a 4.8 ppb aflatoxin-containing ground corn check sample. Three laboratories collected results for the 4.8 ppb aflatoxin check sample each year from 2019 to 2022 until the check sample expired. Not all labs participated in the interlaboratory evaluation each year. The average, SD, RSD, and Z-score are presented for each lab in Table 1. With all laboratory results included, the overall average was 5.7 ppb, although the results from Laboratory 6 deviated significantly from the overall average with a Z-score of 3.1 and results of 11.1 ppb, 11.5 ppb and 43 ppb for extractions of 3 samplings of the same check sample. Since Laboratory 6 results deviated by three standard deviations, along with the large relative standard deviation, those results were removed, and the average and standard deviation recalculated at 4.8 ± 0.9 ppb aflatoxin with a mean RSD of 18%. The overall laboratory mean result agreed with the label claim of 4.8 ± 1.0 ppb aflatoxin for the check sample. Results from laboratories 4, 6, and 9 had relatively high variability with RSDs >20%.

**Figure 1:**
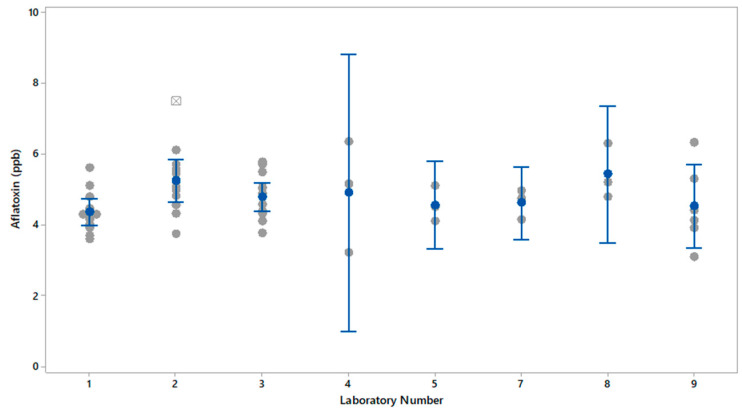
Individual values (gray circles), mean (blue circles), outlier (gray square with a cross) and 95% confidence intervals (blue line and bars) for aflatoxin 4.8 ppb check sample by laboratory.

Table 1 also provides the recalculated Z-scores for each of the laboratories with the results from Laboratory 6 removed. With Laboratory 6 results removed, the Z-scores for the other labs were all within 1 SD from the overall laboratory average. Excluding Lab 6, Figure 1 shows the average, SD, and 95% confidence intervals for the results from each laboratory collected on triplicate extractions for each reference material over 3 years. For Lab 2, the point shown as a square with a cross is a statistical outlier. Excluding Laboratory 6, ANOVA of the mean values for each laboratory was conducted, which showed there was no statistical difference (*p* > 0.05) in the mean values for any of the laboratories.

In 2023, the 4.8 ppb aflatoxin-containing sample had expired, and the check sample was changed to 4.6 ppb aflatoxin-containing ground corn. Only 4 laboratories analyzed the 4.6 ppb aflatoxin sample in 2023, and those results are listed at the bottom of Table 1. The overall laboratory average and SD was 4.9 ± 0.9 ppb aflatoxin with an RSD of 17.6%. An ANOVA of the mean values for each laboratory was conducted, which showed there was no statistical difference (*p* > 0.05) in the mean values for any of the laboratories.

### 3.2. Analytical Results for 19.2 ppb and 21.8 ppb Aflatoxin-Containing Ground Corn Check Samples

Table 2 shows the results each laboratory obtained for the 19.2 ppb and 21.8 ppb aflatoxin-containing ground corn check samples. Three laboratories collected results for the 19.2 ppb aflatoxin check sample each year from 2019 to 2022 until the check sample expired. Average, SD, RSD, and Z-score are provided for each lab in Table 2. With all laboratory results included, the overall average was 20.3 ± 5.7 ppb with an RSD of 28.0%, although Laboratory 6 results again deviated significantly from the overall average with a Z-score of 4.0. Laboratory 6 results were 59 ppb and 27 ppb for extractions of two samplings of the same check sample. This was the same check sample where the other lab overall average was 20.3 ppb. Since Laboratory 6 results deviated by three standard deviations, along with the large relative standard deviation, those results were removed, and the average and standard deviation recalculated. The recalculated lab average and SD were 19.5 ± 2.2 ppb with a mean RSD of 11.5%. The overall laboratory mean result agreed with the label claim of 19.2 ± 2.0 ppb aflatoxin for the check sample. The results from Laboratories 5 and 6 had relatively high variability with RSDs >17%; all other laboratories had RSDs <8%. 

**Figure 2:**
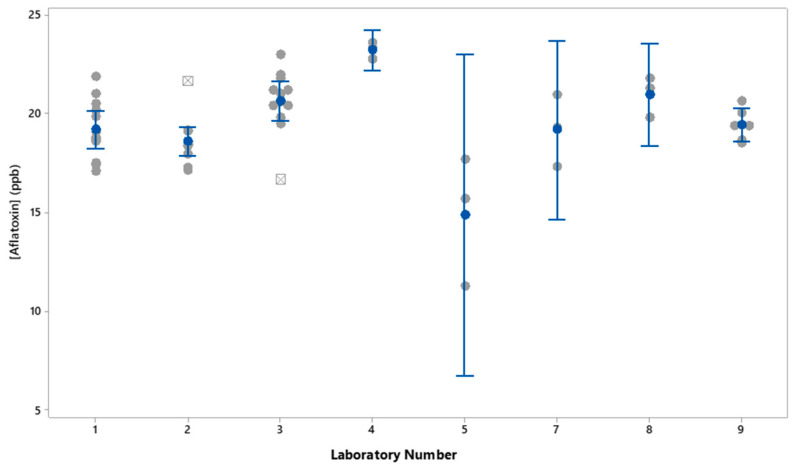
Individual values (gray circles), mean (blue circles), outliers (gray squares with crosses) and 95% confidence intervals (blue lines with bars) for aflatoxin 19.2 ppb check sample by laboratory.

Table 2 also provides the recalculated Z-scores for each of the laboratories with the results from Laboratory 6 removed. With Laboratory 6 results removed, the Z-scores for the other labs were all within 2 SD from the overall laboratory average. Excluding Lab 6, Figure 2 shows the average, SD, and 95% confidence intervals for the results from each laboratory collected on triplicate extractions for each reference material over 3 years. For Labs 2 and 3, the points shown as squares with a cross are statistical outliers. Excluding Laboratory 6, analysis of variance of the mean values for each laboratory showed there were statistical differences (*p* > 0.05) in the mean values for Labs 1, 2, and 9, compared to the mean for Lab 4. Results for Lab 5 were not statistically different from the other lab means due to the RSD of 17.9% and the large confidence interval for Lab 5 results.

In 2023, the 19.2 ppb aflatoxin-containing sample expired, and the check sample was changed to 21.8 ppb aflatoxin containing ground corn. Only four laboratories analyzed the 21.8 ppb aflatoxin sample in 2023, and those results are listed at the bottom of Table 2. The overall laboratory average and SD was 17.7 ± 3.2 ppb aflatoxin with an RSD of 17.8%. An analysis of variance of the mean values for each laboratory was conducted, which showed there was a statistical difference (*p* > 0.05) in the mean values for Labs 2 and 3 compared to the mean for Lab 4. Results for Lab 1 were not statistically different, although the RSD of 20.6% contributed to the lack of a statistical difference.

### 3.3. Analytical Results for a 87.9 ppb Aflatoxin-Containing Ground Corn Check Sample

Table 3 shows the results each laboratory obtained for an 87.9 ppb aflatoxin-containing ground corn check sample. Several laboratories participated in analyzing the sample in various years, although only three laboratories collected results for the 87.9 ppb aflatoxin check sample each year from 2019 to 2023. Average, SD, RSD, and Z-score are provided for each lab in Table 3. With all laboratory results included, the overall average was 97.6 ± 12.4 ppb with an RSD of 12.6%. The overall laboratory mean result was 11% higher than the label claim of 87.9 ± 11.9 ppb aflatoxin for the check sample, although the laboratory mean was within one SD of the label claim. For this check sample, Laboratory 6 results were consistent with the overall mean and had a Z-score of 0.4 so Laboratory 6 results were included in the statistical analysis. Laboratory 5 results were 46.5 ppb, 81.2 ppb and 77.3 ppb for extractions of three samplings of the same check sample. Lab 5 mean had a Z-score of −2.4 compared to the overall mean result for all the labs. Since the results did not deviate more than three SD, the results for Lab 5 were not removed from the analysis. Results from Laboratories 5 and 6 had relatively high variability with RSDs > 19%, while all other laboratories had RSDs < 10%. 

**Figure 3:**
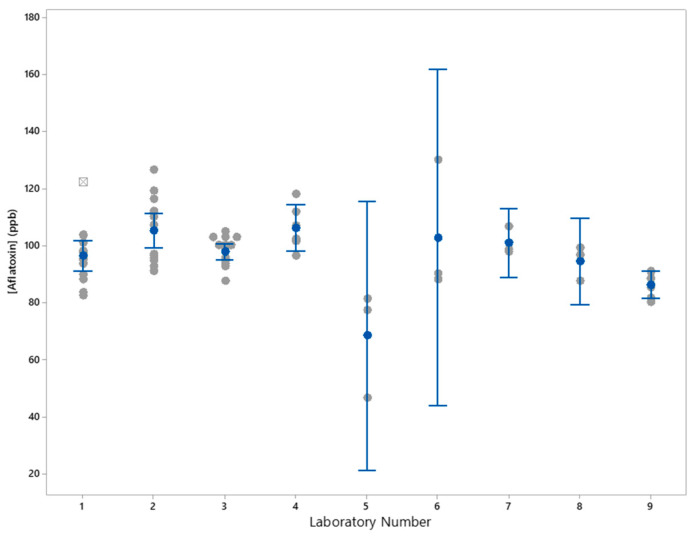
Individual values (gray circles), mean (blue circles), outlier (gray square with cross), and 95% confidence intervals (blue lines with bars) for aflatoxin 87.9 ppb check sample by laboratory.

Figure 3 shows the average, SD, and 95% confidence intervals for the results from each laboratory collected on triplicate extractions for each reference material over 4 years. For Lab 1, the point shown as a square with a cross is a statistical outlier. Laboratories 5 and 6 had RSDs of 22.7% and 18.8%, respectively, which also show in the relatively large 95% confidence intervals shown in Figure 3. Analysis of variance of the mean values for each laboratory showed there was a statistical difference (*p* > 0.05) in the mean value for Lab 9 which was statistically lower than the mean for Lab 2 and Lab 4. Otherwise, there was no statistical difference in the mean for the other labs. Results for Labs 5 and 6 were not statistically different from the other lab means due to the RSD of 22.7% and 18.8% and the large confidence interval for the Lab 5 and Lab 6 results, respectively.

### 3.4. Analytical Results for 25 ng/mL and 1000 ng/mL Aflatoxin Standard Solutions

Table 4 provides experimentally determined results for each laboratory for aflatoxin analytical solutions prepared as described in the Methods section. In 2019 and 2020, analytical solutions were prepared at 25 ng/mL aflatoxin in methanol and provided to the labs. Each year, a different stock solution was prepared. In subsequent years, analytical solutions were prepared at 1000 ng/mL and provided to the labs for analysis. For the 1000 ng/mL aflatoxin standard solution, the overall lab average and SD was 962 ± 72 ng/mL with an RSD of 7.5%. Labs 1 through 4 participated for more than one year in the collection of experimental data for the 1000 ng/mL solutions; Table 4 provides the lab averages along with SD, RSD, and Z-score. The results from Laboratory 9 were outliers and removed from the calculation of the overall lab mean and for the calculation of the Z-score. Except for Lab 5, Z-scores were ≤1.0 for the other labs that collected data for the 1000 ng/mL aflatoxin solution standard. 

## 4. Discussion

Three samples from the same check sample containing 4.8 ppb aflatoxin by label were evaluated by nine analytical laboratories. Laboratory 6 had mean results of 21.9 ppb aflatoxin for the three samples, which deviated significantly from the label and the consensus lab average of 5.7 ppb. Laboratory 6 also had high variability in the results for the three extractions of the same bulk sample, with 68.3% RSD. Laboratory 6 used HPLC/MS/MS for the detection of aflatoxin in the sample and indicated that not all laboratories using HPLC/MS/MS methods obtain accurate and precise results. This is important to consider when comparing results from other methods, such as ELISA and lateral flow to HPLC/MS/MS. Dib et al. reviewed the accuracy and sensitivity of various methods to extract and quantitate mycotoxins in foods [21]. Their work also showed that there can be significant differences in the accuracy and recovery of toxins from food depending on the extraction solvent, extraction procedure, sample type, sample clean-up procedure, and the detection method. Removing the results from Laboratory 6 resulted in an overall lab average of 4.8 ± 0.9 ppb aflatoxin, which agreed with the label claim for the check sample. Other laboratory results did not differ significantly from the expected check sample toxin concentration of 4.8 ppb, which indicated that the inaccuracy and variability in results from Laboratory 6 were not likely due to inhomogeneity of the toxin in the check sample. Not including Laboratory 6, a review of the results from all the other laboratories for this check sample showed that the results ranged from 3.1 to 7.5 ppb aflatoxin. The range in aflatoxin results was due to a combination of variability in the check sample and variability of the methods. The check sample was naturally contaminated with aflatoxin, which can result in variability in the distribution of aflatoxin throughout the sample even though the check sample was ground and well mixed. Laboratories 1, 2, and 3 each analyzed nine replicates of the check sample over the 4-year period. The RSDs were 12.8%, 17.4%, and 12.9%, respectively, which suggests that the variability in distribution of aflatoxin within the check sample is less than 13% since the RSD in laboratory results includes all sources of variability, sample inhomogeneity, toxin recovery, procedure, method, user, instrument, calibration, and standards. With Laboratory 6 removed, ANOVA of the results showed that there was no statistical difference in the mean results for the other laboratories. The 95% confidence interval for the results from Laboratory 4 and Laboratory 8 were larger than the other laboratories. This suggests Laboratories 4 and 8 could improve consistency in results by identifying and reducing sources of variability in the laboratory’s methods or instrumentation.

Comparison of results for triplicate samplings of the 19.2 ppb aflatoxin-containing check sample showed that mean results from Laboratory 6 of 43.0 ppb aflatoxin were again significantly different than the label claim or the consensus laboratory average of 20.3 ppb aflatoxin. Removing the results from Laboratory 6 resulted in a consensus mean of 19.5 ± 2.2 ppb aflatoxin, with a consensus mean RSD of 11.5%. The consensus average agreed with the label claim of 19.2 ± 2.0 ppb aflatoxin for this check sample. Laboratories 5 and 6 had RSDs exceeding the consensus RSD, suggesting that those laboratories could improve the precision of their results by identifying and reducing sources of variability in their methods or instrumentation. With results from Laboratory 6 removed, ANOVA of the results showed that there was a statistical difference in the mean results for Labs 1, 2, and 9 compared to Laboratory 4. Results from Laboratory 4 were significantly higher. There was no statistical difference in the results from Laboratory 5, although that was mainly due to the imprecision of the results for Laboratory 5. The points shown as squares with a cross in Figure 2 are statistical outliers and demonstrate that even for laboratories with normally accurate and precise results, there can be a difference that occurs in replicate sampling that is not consistent with the overall mean. Such differences could be due to sample homogeneity or the method and demonstrate the need for replicate sampling to exclude outliers.

A comparison of the mean results for the 87.9 ppb aflatoxin-containing check sample showed that the mean results for Laboratory 6 of 102.7 ppb aflatoxin were consistent with the consensus mean of 97.6 ppb aflatoxin with a Z-score of 0.4. The consensus laboratory mean result was 11% higher than the label claim of 87.9 ± 11.9 ppb aflatoxin for the check sample, although the laboratory mean was within one SD of the label claim. The 11% difference does demonstrate a difficulty that can occur using naturally incurred mycotoxin check samples as references for other methods such as lateral flow or ELISA, since using this check sample to set a standard curve for those methods could introduce a bias in the result. Laboratory 5 had mean results of 68.3 ppb aflatoxin that deviated from the consensus mean of 97.6 ppb aflatoxin with a Z-score of −2.4. Since the difference was less than three standard deviations from the consensus mean, the results from Laboratory 5 were included in the ANOVA. Variability of results for Laboratory 5 and Laboratory 6 with RSDs of 22.7% and 18.8%, respectively, were also greater than the other laboratories which had RSDs < 10%. Results from Laboratory 9 were statistically lower than results from Laboratories 2 and 4. Otherwise, the mean results for the other laboratories were not statistically different.

In addition to evaluation of the naturally aflatoxin-contaminated check samples, standard aflatoxin solutions were analyzed by each laboratory. Analysis of aflatoxin standard solutions removes variability of the check sample and variability in the recovery of aflatoxin from the results. Deviations from the consensus value of the aflatoxin standard solution for a laboratory indicate an issue with the laboratory’s analytical procedures or instrumentation. Laboratory 9 results for the 1000 ng/mL aflatoxin standard were significantly different than results from the other laboratories, which indicated that there was an issue with the method or instrumentation for that sampling. Trends were detected in results for other laboratories relative to the consensus means, but there were no deviations large enough to remove the results from the analysis. Deviations on the high or low side of the consensus mean can be used by laboratories to determine whether there is a bias in the results.

## Figures and Tables

**Table 1:** Experimentally determined aflatoxin concentration for the 4.8 ppb and 4.6 ppb check samples by laboratory number.

Expected [Aflatoxin] (ppb)		Experimental [Aflatoxin] (ppb) by Laboratory Number								
	Date	1	2	3	4	5	6	7	8	9
4.8	9/19/19	3.6	5.5	4.1						
4.8	9/19/19	4.1	3.8	5.5						
4.8	9/19/19	4.5	5.6	5.7						
4.8	8/1/20	3.9	4.3	5.8				5.0		3.1
4.8	8/1/20	4.3	4.6	5.0				4.2		3.9
4.8	8/1/20	5.6	7.5	4.9				4.8		5.3
4.8	8/21/21	4.4	5.0	5.1					5.2	4.4
4.8	8/21/21	4.8	5.2	4.4					6.3	6.3
4.8	8/21/21	5.1	5.7	4.4					4.8	4.1
4.8	7/1/22	3.7	4.8	3.8	3.2	5.1	11.1			
4.8	7/1/22	4.0	6.1	4.6	5.2	4.5	11.5			
4.8	7/1/22	4.3	5.1	4.3	6.4	4.1	43			
	Ave	4.4	5.3	4.8	4.9	4.6	21.9	4.6	5.4	4.5
	SD	0.6	0.9	0.6	1.3	0.4	14.9	0.3	0.6	1.0
	RSD	12.8%	17.4%	12.9%	26.3%	9.0%	68.3%	7.3%	11.7%	22.8%
	Z-Score	−0.3	−0.1	−0.2	−0.1	−0.2	3.1	−0.2	0.0	−0.2
	Z-Score *	−0.5	0.5	0.0	0.1	−0.3	19.6	−0.2	0.7	−0.3
4.6	11/23/23	4.6	4.0	4.5	4.5					
4.6	11/23/23	5.9	6.0	4.1	5.1					
4.6	11/23/23	3.7	5.7	6.4	4.4					
	Ave	4.7	5.2	5.0	4.7					
	SD	0.8	0.8	0.9	0.3					
	RSD	16.7%	14.9%	17.4%	6.0%					
	Z-Score	−0.3	0.4	0.1	−0.3					

* Z-scores recalculated with results from Lab 6 removed.

**Table 2:** Experimentally determined aflatoxin concentration for the 19.2 ppb and 21.8 ppb check samples by laboratory number.

		Experimentally Determined [Aflatoxin] (ppb) by Laboratory Number								
Label Aflatoxin (ppb)	Date	1	2	3	4	5	6	7	8	9
19.2	9/19/19	18.8	17.1	22.0						
19.2	9/19/19	19.9	18.5	21.0						
19.2	9/19/19	18.6	17.3	23.0						
19.2	8/1/20	17.1	18.5	21.2				19.3		20.6
19.2	8/1/20	17.4	18.6	21.2				17.3		18.6
19.2	8/1/20	17.5	18.4	20.4				21.0		18.5
19.2	8/21/21	21.9	19.1	20.4					21.3	19.4
19.2	8/21/21	21.0	18.6	19.8					19.8	19.4
19.2	8/21/21	20.5	21.7	19.5					21.8	20.1
19.2	7/1/22	19.1	18.4	20.6	23.6	11.3	59			
19.2	7/1/22	20.2	18.0	16.7	23.3	17.7	27			
19.2	7/1/22	18.7	19.2	21.8	22.8	15.7				
	Ave	19.2	18.6	20.6	23.2	14.9	43.0	19.2	21.0	19.4
	SD	1.4	1.1	1.5	0.3	2.7	16.0	1.5	0.8	0.7
	RSD	7.5%	5.9%	7.3%	1.5%	17.9%	37.2%	7.7%	4.1%	3.8%
	Z-Score	−0.2	−0.3	0.1	0.5	−1.0	4.0	−0.2	0.1	−0.2
	Z-Score *	−0.1	−0.4	0.5	1.7	−2.0	10.5	−0.1	0.7	0.0
21.8	11/23/23	20.6	16.2	17.9	21.8					
21.8	11/23/23	13.9	15.4	16.7	21.6					
21.8	11/23/23	13.4	14.6	17.8	22.9					
	Ave	15.9	15.4	17.5	22.1					
	SD	3.3	0.7	0.5	0.6					
	RSD	20.6%	4.3%	3.1%	2.6%					
	Z-Score	−0.6	−0.7	−0.1	1.4					

Z-Score *—Recalculated with results from Lab 6 removed.

**Table 3:** Experimentally determined aflatoxin concentration for the 87.9 ppb check samples by laboratory number.

		Experimentally Determined [Aflatoxin] (ppb) by Laboratory Number								
Label Aflatoxin (ppb)	Date	1	2	3	4	5	6	7	8	9
87.9	9/19/19	100.9	109.9	100.0						
87.9	9/19/19	103.6	107.0	100.0						
87.9	9/19/19	97.5	111.3	103.0						
87.9	8/1/20	82.4	91.0	103.0				97.8		80.0
87.9	8/1/20	83.5	94.7	103.0				98.6		88.5
87.9	8/1/20	88.2	95.5	105.0				106.6		81.6
87.9	8/21/21	93.6	126.6	99.9					96.6	90.6
87.9	8/21/21	95.4	97.0	95.9					99.1	91.0
87.9	8/21/21	94.8	119.2	99.0					87.5	85.3
87.9	7/1/22	103.3	96.3	92.6	106.8	46.5	90			
87.9	7/1/22	98.1	95.5	93.4	118.0	81.2	88			
87.9	7/1/22	89.8	92.6	93.6	102.4	77.3	130			
87.9	11/23/23	96.6	111.3	94.3	111.8					
87.9	11/23/23	93.7	112.0	95.2	101.4					
87.9	11/23/23	122.2	116.2	87.4	96.3					
	Ave	96.2	105.1	97.7	106.1	68.3	102.7	101.0	94.4	86.2
	SD	9.2	10.7	4.8	7.1	15.5	19.3	4.0	5.0	4.2
	RSD	9.6%	10.2%	4.9%	6.7%	22.7%	18.8%	3.9%	5.3%	4.9%
	Z-Score	−0.1	0.6	0.0	0.7	−2.4	0.4	0.3	−0.3	−0.9

**Table 4:** Experimentally determined aflatoxin concentration for analytically prepared aflatoxin solutions.

		Experimentally Determined [Aflatoxin] (ng/mL) by Laboratory Number								
Standard Solution [Aflatoxin] (ng/mL)	Date	1	2	3	4	5	6	7	8	9
25	9/19/19	26.9	28.7	25						
25	8/20/20	24	25	29				24.1		25.4
1000	8/21/21	995	864	1074					907	3654.0 *
1000	7/22/22	991.1	967	914	910	1112.0	1028			
1000	10/23/23	950.8	985.8	891.9	873					
	Ave	979.0	938.9	960.0	891.5	1112.0	1028.0		907.0	
	SD	20.0	53.5	81.1	18.5					
	RSD	2.0%	5.7%	8.5%	2.1%					
	Z-Score	0.2	−0.3	0.0	−1.0	2.1	0.9		−0.8	

* Result was a statistical outlier and not included in overall lab average.

## Data Availability

The original contributions presented in the study are included in the article, further inquiries can be directed to the corresponding author.
